# Kynurenic Acid: The Janus-Faced Role of an Immunomodulatory Tryptophan Metabolite and Its Link to Pathological Conditions

**DOI:** 10.3389/fimmu.2017.01957

**Published:** 2018-01-10

**Authors:** Elisa Wirthgen, Andreas Hoeflich, Alexander Rebl, Juliane Günther

**Affiliations:** ^1^Institute for Genome Biology, Leibniz Institute for Farm Animal Biology, Dummerstorf, Germany

**Keywords:** kynurenic acid, immunomodulation, inflammation, aryl hydrocarbon receptor, G-protein-coupled receptor 35, tryptophan metabolism, microbiota

## Abstract

Tryptophan metabolites are known to participate in the regulation of many cells of the immune system and are involved in various immune-mediated diseases and disorders. Kynurenic acid (KYNA) is a product of one branch of the kynurenine pathway of tryptophan metabolism. The influence of KYNA on important neurophysiological and neuropathological processes has been comprehensively documented. In recent years, the link of KYNA to the immune system, inflammation, and cancer has become more apparent. Given this connection, the anti-inflammatory and immunosuppressive functions of KYNA are of particular interest. These characteristics might allow KYNA to act as a “double-edged sword.” The metabolite contributes to both the resolution of inflammation and the establishment of an immunosuppressive environment, which, for instance, allows for tumor immune escape. Our review provides a comprehensive update of the significant biological functions of KYNA and focuses on its immunomodulatory properties by signaling *via* G-protein-coupled receptor 35 (GPR35)- and aryl hydrocarbon receptor-mediated pathways. Furthermore, we discuss the role of KYNA–GPR35 interaction and microbiota associated KYNA metabolism for gut homeostasis.

## Biological Significance of the Kynurenine Pathway (KP)

**Figure 1:**
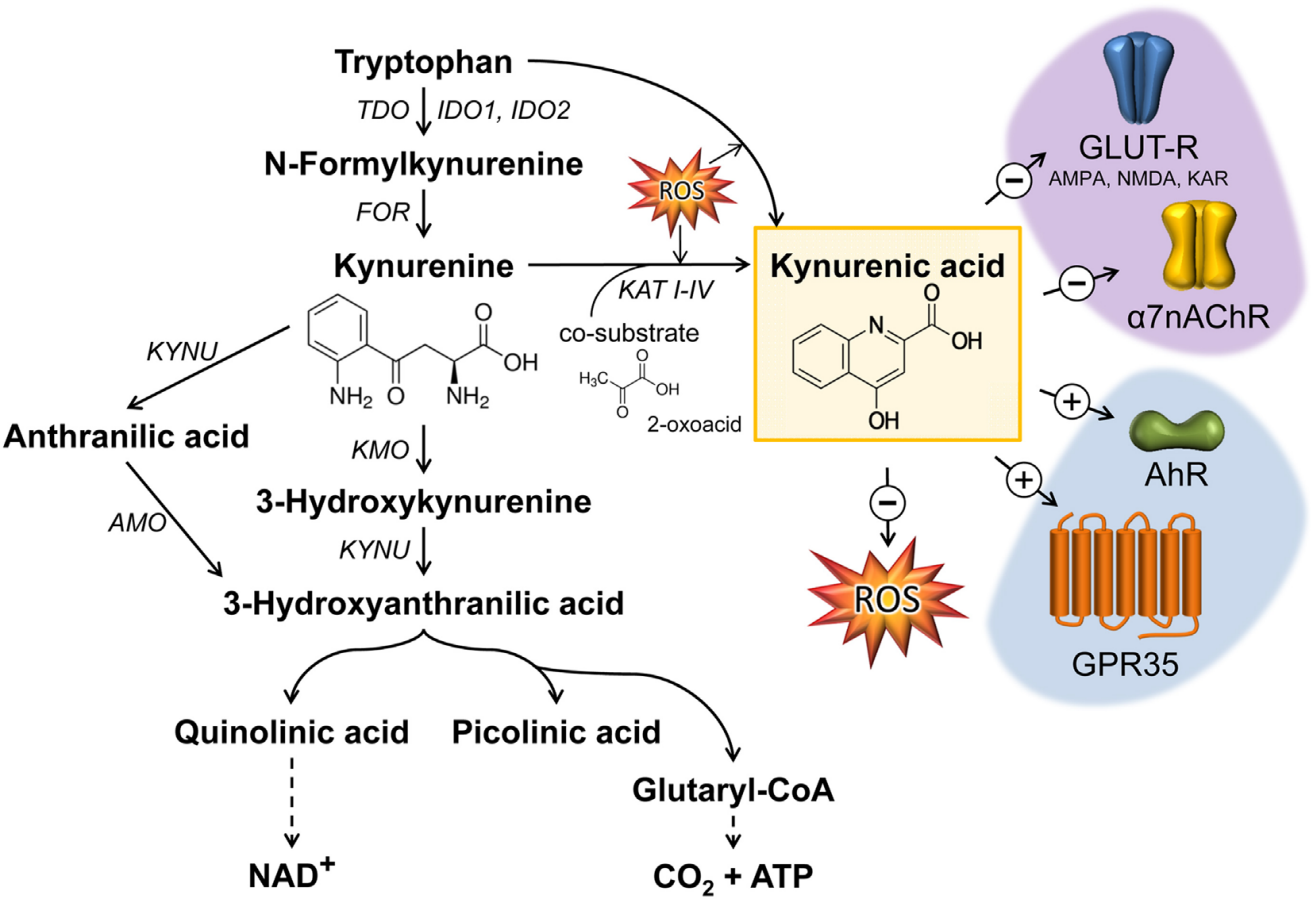
Kynurenic acid (KYNA) synthesis along the kynurenine pathway and its mode of action. The catabolism of TRP by the enzymes TDO or IDO represents the rate-limiting step in KYNA synthesis. The intermediate metabolite kynurenine can be further processed through three distinct pathways to form KYNA, 3-hydroxykynurenine, and anthranilic acid. KYNA is formed by the irreversible transamination of KYN either *via* kynurenine aminotransferases (KAT I–IV) or through the action of reactive oxygen species (ROS). KYNA is a non-competitive antagonist of ionotropic glutamate receptors (GLUT-R) as well as of the α7 nicotinic acetylcholine receptor (α7nAChR) expressed on neuronal cells. Apart from neuromodulatory properties, KYNA is an agonist of the broadly expressed G-protein-coupled receptor 35 (GPR35) and aryl hydrocarbon receptor (AhR). Furthermore, KYNA functions as an ROS scavenger. Black arrows mark enzymatic reactions and dashed arrows include more than one catalytic reaction step. FOR, formamidase; IDO, indolamine 2,3-dioxygenase; TDO, tryptophan 2,3-dioxygenase; TPH, tryptophan hydroxylase; KAT, kynurenine aminotransferase; KMO, kynurenine 3-monooxygenase; KYN, kynureninase; AMO, anthranilate 3-monooxygenase; AMPA, α-amino-3-hydroxy-5-methyl-4-isoxazolepropionic acid receptor; NMDA, *N*-methyl-D-aspartate receptor; KAR, kainate receptor.

The degradation of tryptophan (TRP) along the KP plays a crucial role in the regulation of the immune response, notably as a counter-regulatory mechanism in the context of inflammation (1–3). An overview of the KP is presented in Figure 1. Three rate-limiting enzymes of KP, tryptophan 2,3-dioxygenase (TDO) and indolamine 2,3-dioxygenase (IDO) 1 and 2, have been described in the literature thus far. TDO is positively regulated by TRP in order to maintain the homeostasis of TRP (4, 5). Furthermore, the expression and activity of TDO is regulated by hormones such as cortisol, insulin, glucagon, or epinephrine (6–8). IDO1 and 2 are upregulated by inflammatory stimuli such as interferon-γ (IFN-γ) (9–14). The significance of KP activation depends on the production of biologically active metabolites such as kynurenine (KYN), kynurenic acid (KYNA), quinolinic acid (QUIN), or anthranilic acid mediating various immuno- and neuromodulative functions. Within the central nervous system, it has been well documented that metabolites such as KYNA and QUIN modulate neurological functions. Thus, KYNA acts as an antagonist affecting all ionotropic glutamate receptors including NMDA, AMPA, and kainate receptors as well as the α7 nicotinic acetylcholine receptor (α7nAChR) assuming it as a neuroprotective metabolite (15–18). However, the inhibition of α7nAChR by KYNA is extensively debated because some later studies addressing this mechanism (3, 19–21) could not recapitulate the original results from Hilmas et al. (18). Dysregulation of KP, resulting in alterations of the balance between KYNA and QUIN, has been described in many neurological disorders (22). However, alterations of KYNA are also described in several inflammatory-related states, such as sepsis or inflammatory bowel disease (IBD), and are discussed as a potential marker in cancer patients (23–25). It is generally accepted that KYNA mediates immunosuppressive effects (22), notably by targeting the G-protein-coupled receptor 35 (GPR35)- or aryl hydrocarbon receptor (AhR)-associated signaling pathways (2, 26, 27).

## Endogenous KYNA Synthesis and Its Inflammatory Regulation in Vertebrates

Generation of KYNA was described in endothelial cells (28), epithelial cells (29, 30), fibroblasts (31), pancreatic islet cells (32), human peripheral blood mononuclear cells (33), skeletal muscle cells (34), and red blood cells (35). Under physiological conditions, KYNA is produced by kynurenine aminotransferases (KATs), which catalyze the irreversible transamination reaction between l-KYN and 2-oxoacid, as a co-substrate, to form KYNA (36, 37), or in the presence of reactive oxygen species (ROS) as illustrated in Figure 1. Currently four proteins named KAT I–IV are described in mammals (38–43). Mediating overlapping biological functions, the mammalian enzymes KAT I and KAT III share high homologies in sequence and genomic structure (42). KAT activity was described in various tissues, such as liver, kidney, small intestine, dermal fibroblasts, and brain (31, 38, 44, 45). In human cardiac muscle, there is evidence that the KAT system differs from brain KAT regulation regarding optimum pH, co-substrate specificity, and sensitivity to inhibition by amino acids such as l-TRP (36). Regarding the inflammatory regulation of KATs, there is no consensus, assuming the existence of cell type-dependent regulatory differences. Studies in human dermal fibroblasts reveal that tumor necrosis factor α (TNF) alone does not affect the number of transcripts, whereas IFN-γ alone (or in combination with TNF) decreases the transcript abundance of KAT I, III, and IV after 48 h. Due to the fact that in this study the metabolite concentration of KYNA was increased in supernatants the decrease of KATs 48 h after cytokine stimulation may reflect a negative feedback mechanism (31). In fetal astrocytes, IFN-γ increases the transcript levels of KAT I and II after 24 h (46), whereas no effect of IFN-γ was observed in neuronal cells (47). In mice, an intraperitoneal (i.p.) lipopolysaccharide (LPS) challenge increased KAT-I mRNA expression in the hippocampus 6 h postchallenge, followed by a decrease after 24 h, whereas KAT-II mRNA expression was decreased 24 h after LPS treatment (48).

In addition to KAT-catalyzed KYNA synthesis, alternative routes for KYNA synthesis in the presence of ROS have been described (49) (Figure 1). Thus, it is assumed that indole-3-pyruvic acid, a reaction product of tryptophan-2-oxoglutarate-induced transamination of TRP, undergoes pyrrole ring cleavage followed by a spontaneous cyclization generating KYNA (50). Furthermore, l-KYN can be converted to KYNA in the presence of hydrogen peroxide (51) or KYNA formation can result from reactions of KYN or indole-3-pyruvic acid under conditions generating free radicals (52, 53).

Elevations of KYNA blood concentrations were experimentally induced under different inflammatory conditions. In pigs, the i.p. application of LPS increased plasma levels of KYNA. However, the *ex vivo* LPS stimulation of whole blood culture failed to elevate KYNA in supernatants (54), assuming sources of KYNA production other than blood cells or the requirement of additional inflammatory mediators, which are not produced in blood cells. Increased KYNA plasma concentrations were also detected in mice that were repeatedly stressed. In this context, the elevated KYNA levels were proposed to be induced by a systemic low-grade inflammation due to an altered intestinal barrier function (55). Interestingly, in this study the application of the IDO inhibitor l-1-methyltryptophan (1-MT), a TRP analog, specifically increased plasma concentrations of KYNA by a yet unknown pathway (55).

## KYNA Degradation and Excretion

Kynurenic acid is described as one of the end products of KP in animals, assuming no uptake or further metabolism of KYNA. In a study including different rodent species, 90% of radioactively labeled KYNA was excreted in urine within 24 h of i.p. application (56). Thus, 80–100% of labeled KYNA was excreted unchanged and only small amounts of quinaldic acid and quinaldylglycine were detected (0.3 and 5%, respectively). This is supported by studies in rats, finding that radioactively labeled KYNA was eliminated rapidly after intracerebroventricular microinjection and substantial amounts of radioactivity were recovered in urine 30 min after injection (57). Studies in rabbits described differences in KYNA metabolism depending on the type of administration (58). After oral administration of KYNA, the majority of the dose was detected in the form of quinaldic or 8-hydroxyquinaldic acid, indicating a dehydroxylation of the molecule. In contrast, after subcutaneous administration, 99% of KYNA was recovered unchanged, indicating that the dehydroxylation occurs in the gastrointestinal tract (58) most probably by the gut microbiota. This is supported by the finding that approximately 30% of ingested KYNA was excreted in urine as quinaldic acid in humans (59). The assumption that KYNA is metabolized by microorganisms is supported by the finding that extracts of *Pseudomonas* spp. and *Aerococcus* spp. were able to enzymatically partially degrade KYNA (60, 61).

## Immunomodulative Properties of KYNA

In recent years, numerous *in vivo* and *in vitro* studies have been directed toward the immunomodulatory functions of KYNA. There are strong indications that the action of KYNA varies depending on whether inflammatory or homeostatic conditions are considered. Under homeostatic conditions, KYNA induced interleukin 6 (IL6) mRNA expression 2 h after treatment in the breast cancer cell line MCF-7 (27) and cytokine secretion (TNF, IL6, IL1β, and IL10) in primary murine splenocytes after 72 h (62). A further *in vitro* study indicated that KYNA may be an early mediator of leukocyte recruitment, acting by triggering the activation of neutrophils as well as the adhesion of monocytes to fibronectin and intercellular adhesion molecule 1 *via* β1-/β2 integrin (63). In contrast, KYNA treatment decreased the mRNA expression of IL6 after 6 h in the rat mast cell line RBL-2H3 followed by a return to baseline level after 24 h (64). In addition, 24-h KYNA treatment under homeostatic conditions did not influence IL6 or TNF secretion in the murine microglial cell line BV-2 (65). At first glance, all these findings seem to be very inhomogeneous. However, they clearly demonstrate that knowing that mRNA expression of pro-inflammatory cytokines is normally tightly controlled by mRNA decay and cytokine secretion measurement needs sufficient accumulation time, it is very important for interpretation of such results to know at which time what (mRNA expression or cytokine secretion) was analyzed. In conclusion, these studies, analyzing the effect of KYNA under non-inflammatory conditions, suggest a time- and/or cell type-dependent influence of the treatment.

The KYNA effect under inflammatory conditions appears to be more uniformly. Several *in vitro* studies, using various primary or immortalized leukocyte cell types, have revealed that KYNA can attenuate inflammation elucidated by different stimuli (e.g., LPS). For instance, KYNA reduces TNF expression and secretion (26, 55, 65, 66) and diminishes high-mobility group box 1 (HMGB1) protein secretion in monocytes (66, 67). Likewise, KYNA has been shown to inhibit the secretion of α-defensin HNP1–3 in granulocyte cultures (66) and reduce interleukin 4 release in T-cell receptor stimulated invariant natural killer-like T cells (iNKT) (68). Recently, Elizei et al. (69) demonstrated that KYNA reduced LPS-induced IL23 expression of dendritic cells and inhibited Th17 cell differentiation *in vitro*. The downregulation of the IL23/IL17 axis is known to be beneficial for anti-inflammatory treatment of many immune-mediated diseases (70).

These anti-inflammatory effects of KYNA, frequently observed in many cell models, were confirmed by *in vivo* studies in mice and dogs. For example, KYNA treatment inhibited the LPS induced increase of TNF and nitric oxide (NO) in mice serum and also drastically reduced LPS-induced death in those animals (67). Leukocytes of KYNA-treated mice also exhibited a reduced release of TNF in response to an *ex vivo* LPS challenge (55). Moreover, the increased mucosal leukocyte accumulation and the xanthine oxidoreductase activity, a predominant marker of mucosal superoxide radical production, in the gastrointestinal tract of dogs with experimental colon obstruction were reduced by KYNA treatment (71).

**Figure 2:**
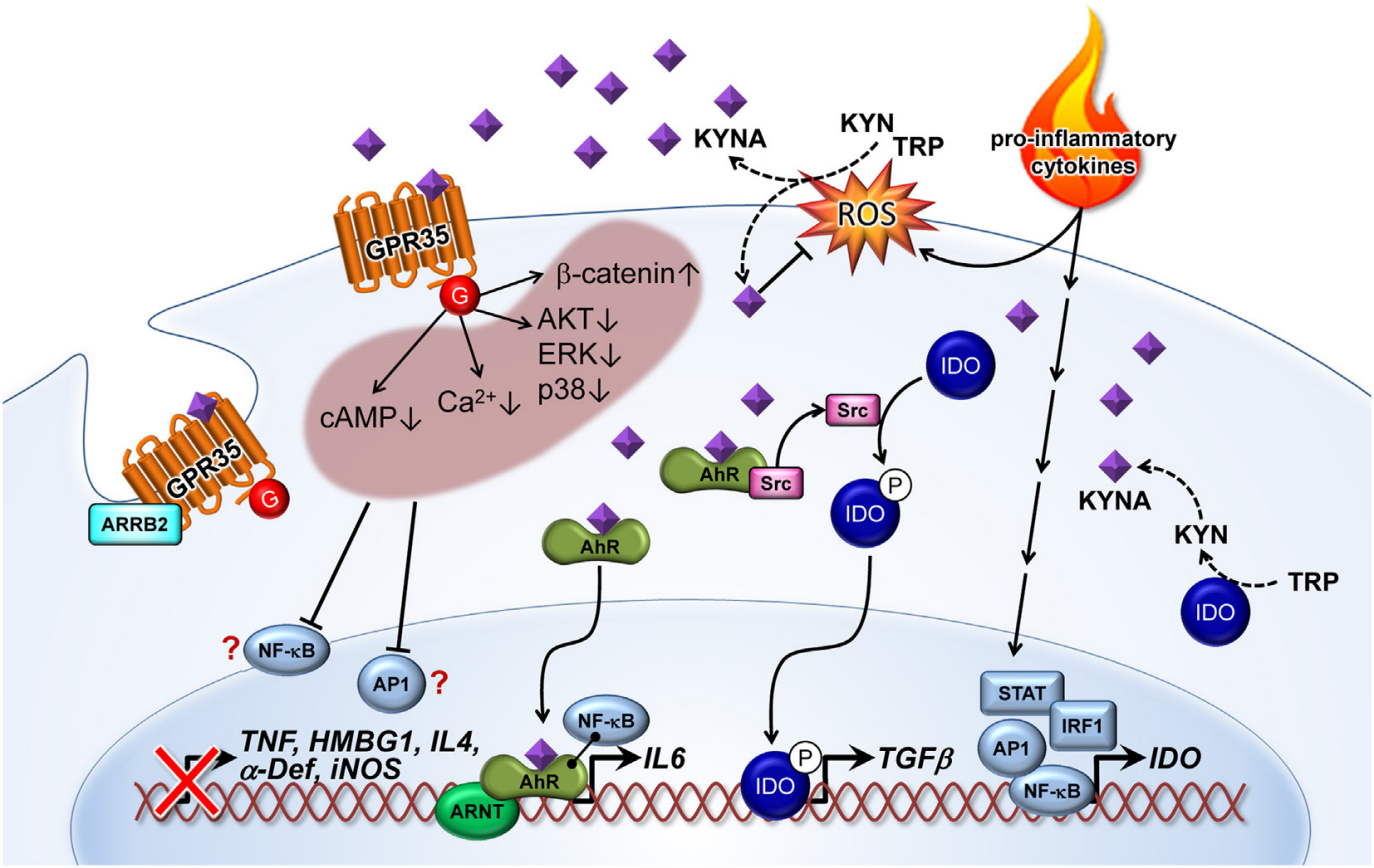
Kynurenic acid (KYNA)-mediated pathways of inflammatory signaling. Pro-inflammatory cytokines induce the expression of indolamine (IDO) enzyme *via* STAT, AP1, IRF1, and NF-κB transcription factor activation. KYNA is formed by the IDO-dependent canonical pathway or by an alternative route through direct kynurenine (KYN) or tryptophan (TRP) transformation by reactive oxygen species (ROS). On the other hand, KYNA as a free radical scavenger decreases ROS level. KYNA binds and activates G-protein-coupled receptor 35 (GPR35)-reducing cAMP and calcium (Ca^2+^) levels in cells. Activation of GPR35 by KYNA may also inhibit phosphorylation of protein kinase B (AKT), extracellular signal-regulated kinase (ERK), and p38 mitogen-activated protein kinase (p38), as well as increasing the level of β-catenin. All of these cellular responses probably decrease activation of relevant inflammatory transcription factors, such as NF-κB and AP1. Therefore, reduced induction of tumor necrosis factor α (TNF), high-mobility group box 1 (HMBG1), interleukin 4 (IL4), α defensin (α-Def), and inducible nitric oxide synthase (iNOS) have frequently been observed in response to KYNA treatment. Recruitment of arrestin β2 (ARRB2) to GPR35 is necessary for internalization and desensitization of the KYNA-activated receptor. Binding of KYNA to the aryl hydrocarbon receptor (AhR) receptor leads to recruitment of the AHR nuclear translocator (ARNT) and induction of IL6 expression. Interaction of the KYNA–AhR complex with NF-κB may also be involved in the induction of IL6. Furthermore, ligand-activated AhR initiates the proto-oncogene tyrosine-protein kinase Src activation and, thereby, the phosphorylation (P) of IDO. Phosphorylated IDO induces the expression of transforming growth factor β1 (TGFβ). NF-κB, nuclear factor κ-light-chain-enhancer of activated B cells; AP1, activator protein 1; STAT, signal transducer and activator of transcription; IRF, interferon-regulatory factor; G, G protein.

Kynurenic acid is a ligand of the GPR35 (26) and the AhR (27, 72) (Figures 1 and 2). The affinity of KYNA for both receptors is in the low micromolar range. However, in various inflammatory and tumor diseases high levels of this metabolite are produced, so it is not surprising that under these conditions KYNA levels are sufficient to activate these receptors (72). In addition to GRP35- and AhR-mediated signals, KYNA has a relevant role as an antioxidant and ROS scavenger (73, 74) (Figures 1 and 2). This indicates that KYNA also actively prevents tissue damage triggered by overshooting inflammation. In addition, induction of the KYNA-synthesizing branch of TRP metabolism may also be relevant for the synthesis of other TRP metabolites, such as serotonin or melatonin. Serotonin and melatonin are known immune regulators whose decrease may influence immune response (75, 76). KYNA synthesis may decrease their abundance either simply by the reduction of the necessary substrate TRP, or by direct inhibition of their synthesis, or induction of their degradation. In this regard, it was found that furafylline-mediated inhibition of CYP1A2, a “classic” AhR-inducible gene, increased 6-hydroxymethylation of melatonin in rat liver slides. This result indicates that AhR signaling, perhaps triggered by KYNA, may be relevant in melatonin catabolism (77).

## KYNA as Agonist of GPR35

G-protein-coupled receptor 35 is expressed in various subpopulations of immune cells, including peripheral monocytes (26), mast cells, basophils, eosinophils (78), and iNKT cells (68). A high level of GPR35 expression was detected throughout the digestive tract (26, 79), as well as in lung, skeletal muscle, uterus, and dorsal root ganglion (79). Moderate expression was found in heart, liver, bladder, spinal cord, whole brain, and cerebrum (79).

Recently, it was found that GPR35 is a high-affinity receptor for the mucosal chemokine CXCL17 (80). Nevertheless, KYNA was the first reported agonist ligand for GPR35. This was identified by high-throughput screening using changes of intracellular calcium (Ca^2+^) in the Chinese hamster ovary cell line, CHO, co-expressing GPR35 and a G-protein mixture as a readout (15). Further in-depth studies revealed that KYNA–GPR35 interaction inhibited N-type Ca^2+^ channels in sympathetic neurons (81) and reduced the plateau phase of ATP-induced calcium transients in astrocytes (82). The later study also demonstrated that KYNA-mediated GPR35 activation decreased forskolin-induced cAMP elevation. Furthermore, the recruitment of β-arrestin 2 mediated GPR35 internalization upon KYNA activation, which led to receptor desensitization (82, 83).

Kynurenic acid may also have an inhibitory effect on the phosphoinositide 3-kinase (PI3K)/protein kinase B (Akt) and mitogen-activated protein kinase (MAPK) pathways. Walczak et al. (84) demonstrated that KYNA decreased phosphorylation of extracellular signal-regulated kinases (ERK) 1/2, p38 MAPK, and Akt in colon epithelial cells. They also found indications that KYNA induced accumulation of β-catenin. MAPK, PI3K/Akt and β-catenin pathways are well-known targets of GPR signaling (85, 86).Therefore, it is possible that the observed inhibition of ERK and p38, as well as the induction of β-catenin accumulation after KYNA treatment, are a consequence of GRP35 activation. Interestingly, all of these described effects of KYNA–GPR35 signaling might lead to the suppression or limitation of inflammation. Increased intracellular calcium is associated with inflammatory signal secretion (87, 88) and triggers the activation of NF-κB (89), which is an essential transcription factor in inflammation (90). The cAMP pathway is known to regulate innate and adaptive immune cell activities (91) [e.g., T-cell functions (92)]. In this respect, there is strong evidence that KYNA–GPR35-mediated inhibition of adenylate cyclase is causal for the downregulation of the IL23/IL17 immune axis observed after KYNA treatment (69). Furthermore, the PI3K/Akt pathway and MAPK’s play crucial roles in generating an inflammatory response (93, 94). Conversely, the β-catenin signaling pathway is known to inhibit inflammation through limiting NF-κB activation by stabilizing the NF-κB inhibitory IκB-factors (95).

## KYNA as Agonist of AhR

Aryl hydrocarbon receptor is a ubiquitously expressed promiscuous ligand-operated receptor of KYNA (27), mediating crucial effects on the regulation of the immune response (96). When binding to a ligand, AhR dimerizes with the AhR nuclear translocator (ARNT) and acts as a transcription factor.

Several studies using AhR knockout mice indicated that this receptor has an important immune regulatory role in inflammation. For instance, AhR-deficient mice have a high susceptibility to LPS-induced septic shock (97) and developed a stronger response after local inflammatory challenge (or insult) in the lung (98, 99). AhR activation is involved in preventing an overshooting pro-inflammatory cytokine induction in response to an inflammatory stimulus in various cells including fibroblasts, endothelial cells, and macrophages (97, 100, 101). Therefore, it has been proposed that a clinical treatment with an appropriated AhR ligand like KYNA may offer a promising therapeutic intervention in inflammatory disorders. Interestingly, brains of AhR knockout mice exhibited an increased KAT-II expression and a higher KYNA level in the cerebral cortex and striatum that is associated with protection against oxidative stress induced by an excitotoxic insult *via* intrastriatal application of QUIN (102). This may be either a result of KYNA-mediated counteraction of NMDAR activation by QUIN, which is known to mediate exitotoxic properties, or of the receptor-independent antioxidative properties of KYNA discussed above. Furthermore, the data of this study indicated a negative feedback-loop between AhR, KAT II, and the AhR ligand KYNA in the brain. Unfortunately, there are no data regarding KYNA levels and KAT expression outside the brain of AhR-deficient mice. Therefore, further analyses are necessary to prove whether this is only a tissue-specific observation or a general finding. However, studies using AhR-deficient mice need to be carefully evaluated because, although these animals appeared relatively normal, this knockout influences several physiologic processes in the animals including structure of the central nervous system and blood cell differentiation (103).

In addition to KYNA, other TRP metabolites, such as the KYNA precursor KYN, have been shown to be ligands of the AhR (104). In humans, pigs, and mice KYNA normally have 3- to 10-fold lower plasma concentrations than KYN (54, 55, 105, 106). However, KYNA is described as a more potent AhR ligand (27) and has a higher stability than KYN (56). It was speculated that AhR interaction with TRP metabolites contributed to immune homeostasis during endotoxin tolerance by activating immunomodulatory signaling (104). The data of Bessede et al. indicated that AhR-associated Src activity, triggered by TRP metabolites bound to AhR, was responsible for IDO1 phosphorylation. These studies revealed that the TRP metabolite KYN, without the IDO1 protein, was insufficient to induce TGFβ expression. The authors speculated that IDO1 phosphorylation represented an independent signaling pathway necessary for TGFβ-mediated immune tolerance. Whether the TRP metabolite KYN or KYNA could be the relevant AhR ligand mediating those immunosuppressive effects remains unclear. It has been demonstrated that KYNA ligated to AhR induced IL6 mRNA expression in breast cancer cells. These authors also showed that a combination of KYNA and pro-inflammatory IL1 induced IL6 much more strongly than either of these factors alone. This synergistic activation of IL6 could be mediated by direct interaction of AhR–ARNT and the NF-κB factor RELB. NF-κB factors are known to be activated by pro-inflammatory cytokines, such as IL1. The role of AhR-NF-κB cross talk was previously described for IL8 gene expression (107). IL6 features pleiotropic activities (108). Although IL6 plays essential roles in promoting inflammation, it also has many anti-inflammatory and regenerative activities (109) reviewed in Ref. (110, 111). Therefore, it is difficult to estimate if KYNA-mediated IL6 expression contributes only to the immunosuppressive function of KYNA. However, studies have shown that IL6 is involved in the development of many chronic inflammatory and cancer diseases (111). For example, the study that demonstrated a KYNA-mediated induction of IL6 in breast cancer cells discussed this observation as part of the mechanisms allowing tumor cells to escape immune surveillance (27). A further interesting point is that IL6 can induce IDO1 *via* STAT3 activation. This signaling is known as the AhR–IL6–STAT3 loop, which is associated with poor prognosis in lung cancer (112). There is increasing evidence that the interaction of AhR with metabolites of the KP, such as KYNA, is relevant for maintaining the immunosuppressive microenvironment in many cancer types (72, 113). The probable mode of action is a TGFβ- and IL6-mediated suppression of T-cell response by interfering with differentiation and activation of regulatory T cells. This is still supported by the fact that various cancer cells secrete KYNA (Table 1).

**Table 1:** Alteration of kynurenic acid (KYNA) metabolism in several pathological states in humans.

Disease	Perturbation of KYNA level	Matrix	Source
**Inflammation-related diseases**			
Multiple sclerosis	Increased vs. healthy controls	Plasma	(114)
Inflammatory bowel disease	Increased vs. healthy controls	Plasma	(23)
Septic shock patients with acute kidney injury	Increased in non-survivor vs. survivors	Plasma	(24)
Out-of-hospital cardiac arrest	Increased in patients with 12-month poor outcome	Plasma	(115)
Rheumatoid arthritis	Decreased vs. patients with osteoarthritis	Synovial fluid	(116, 117)
	Positive correlation with plasma fibrinogen		
	Positive correlation with morning stiffness and pain score	Serum	
Type 2 diabetes	Increased vs. healthy control	Plasma	(118)
Chronic kidney disease	Increased with severity stage	Serum	(119)
Odontogenic abscesses	Increased vs. healthy subjects	Saliva	(120)

**Cancer**			
Colon carcinoma	Increased vs. non-carcinoma cells	Supernatant	(30)
Adenoma tubovillosum and A. tubulare	Increased vs. non-carcinoma cells	Supernatant	(30)
Non-small cell lung cancer	Increased vs. healthy controls	Serum	(121)
	Increased in patients with metastatic spread to lymph nodes vs. non-metastatic patients		
Prostate cancer	Decreased vs. participants without malignancy	Urine	(25)
Primary cervical cancer	Decreased vs. healthy controls	Serum	(122)
Glioma	Decreased vs. healthy controls	Serum	(123)

**Mental disorders**			
Affective psychosis	Decreased vs. healthy controls	Serum	(124)
Chronic schizophrenia	Decreased vs. healthy control	Serum	(125)
Chronic migraine	Decreased vs. healthy controls	Serum	(126)
Cluster headache	Decreased vs. healthy controls	Serum	(127)
Alzheimer’s type dementia	Positive correlation of KYNA with cognitive function	Plasma	(128)
Schizophrenia with distress intolerance	Increased vs. patients with distress tolerance and healthy controls, positive correlation with severity of clinical symptoms	Saliva	(129)
Schizophrenia	Increased vs. heathy controls	CSF	(130–132)
Alzheimer’s dementia	Decreased vs. healthy control	Plasma, red blood cells	(35)
Alzheimer’s disease	Positive correlation with P-tau and soluble intercellular adhesion molecule-1	CSF	(37)

**Inherited diseases/diseases with questionable cause**			
Down syndrome	Increased vs. control specimens	Temporal cortex, urine	(133, 134)
Huntington’s disease	Reduced vs. healthy controls	Brain areas, CSF	(135, 136)
Amyotrophic lateral sclerosis (ALS)	Increased patients with bulbar onset and severe clinical status of ALS vs. healthy control	CSF	(137)
	Decreased in patients with severe clinical status of ALS vs. healthy control	Serum	
Irritable bowel syndrome	Decreased vs. healthy controls	Plasma, serum	(138, 139)

Irrespective of the presumed KYNA-mediated tumor-immune escape, another research group found that high KYNA concentrations inhibit proliferation and migration of cancer cell lines *in vitro* (84, 140, 141). This seems to be mediated by interference with the cyclin-dependent kinase inhibitor p21(WAF1/CIP1) pathway (142). All this indicates that KYNA can act as both tumor-promoting as well as tumor-inhibiting factor. However, all mentioned studies used cancer cell lines and characterized the effect of KYNA under *in vitro* conditions. Therefore, it must be kept in mind that further studies are needed to validate the KYNA effects on tumor development.

## KYNA and Its Link to Pathological Conditions

Alterations of KYNA metabolism in both periphery and brain are described for several pathological states in humans (Table 1).

### Inflammation-Related Diseases

Kynurenic acid levels are increased in peripheral blood of patients suffering from type 2 diabetes, multiple sclerosis, IBD, and chronic kidney disease (23, 114, 118, 119) as well as in saliva of patients suffering from odontogenic abscesses (140). Regarding these chronic inflammatory conditions, it remains unclear whether the elevation of KYNA is either a compensatory response due to inflammatory signaling or a primary abnormality, inducing specific patterns of diseases. However, it is presumed that chronic stress or low-grade inflammation may induce the production of KYNA (55, 118), provoking various immunomodulative actions due to KYNA-mediated signaling pathways. Contrary to elevated peripheral KYNA levels in IBD, blood levels of KYNA were found to be decreased in patients with irritable bowel syndrome (IBS), which is—in contrast to IBD—a functional gastrointestinal disorder without chronic inflammation (138, 139). However, KYN was increased in plasma of patients with IBS (138) and with severe IBS (143). These data may indicate an inflammatory induction of IDO. However, in this study there was no evidence for inflammatory processes, such as increased serum levels of IFN-γ or TRP depletion compared with healthy controls. Christmas et al. (139) reported decreased levels of KYNA, KYN, and 3-hydroxyanthranilic acid in IBS. This provides evidence for a generally inhibited TRP degradation, resulting in a reduced TRP oxidation. It was assumed that the increased free TRP would be a source for utilizing serotonin, which may increase gut secretions and motility as described in diarrhea-predominant IBS.

Studies of patients with septic shock and acute kidney injury showed that a failed reduction of KYNA after a hemofiltration treatment might predict fatal outcomes (24). The authors assumed that the increased plasma levels of KYNA depended on the rate of KYNA synthesis and not on a failed renal excretion since KYNA and other TRP metabolites were eliminated continuously in these patients by hemodialysis. An increased KP activation, for instance, measured by increased KYN and KYNA levels, was observed in non-survivors of out-of-hospital cardiac arrest during early and late stage of disease (115). In this context, it is possible that increased generation of KP metabolites may reflect an overshooting pro-inflammatory response, which subsequently provokes the establishment of a protracted immunoparalysis as described in sepsis (2).

A positive correlation of serum KYNA with morning stiffness and pain score was found in patients with rheumatoid arthritis (RA) (116), indicating that an increased level of inflammation is correlated with increased circulating KYNA levels. This assumption is supported by the finding that KYNA concentrations in the synovial fluid of RA patients were positively correlated with plasma fibrinogen (116), which is described as a marker for disease activity reflecting the acute phase response (144). *In vitro* studies revealed that KYNA inhibited the proliferation of synoviocytes and enhanced the antiproliferative action of drugs, targeting the prevention of hyperplasia of synovial fibroblasts (145). In patients with RA, KYNA was decreased compared with patients with osteoarthritis (OA; no inflammatory background). This was in accordance with the results of an earlier study revealing that KYNA was decreased in RA compared with OA, while IDO activity was increased, which is not surprising due to RA-induced inflammation (117). According to the described antiproliferative function of KYNA on synovial fibroblasts, impaired KYNA synthesis may provoke the development of hyperplasia in RA.

### Cancer

Increased concentrations of KYNA were detected in supernatants of colon-derived cells from patients diagnosed with colon carcinoma, adenoma tubulovillosum, or adenoma tubulare compared with a healthy control group (30). Furthermore, KYNA was elevated in the serum of patients with non-small cell lung cancer compared with healthy volunteers and increased in patients with metastases that spread to lymph nodes vs. non-metastatic patients (121). These findings support the suggestion that many cancer types secrete KYNA provoking the establishment of an immunosuppressive microenvironment (72, 113). In contrast, KYNA was decreased in the serum of patients suffering from glioblastoma compared with healthy controls (123). Due to the increased ratio of KYN to TRP, an indicator for IDO activity, an over-activation of KP was postulated. However, the plasma concentrations of TRP, KYN, KYNA, and QUIN were decreased compared with healthy controls, indicating a depletion of TRP by other mechanisms. Indeed, Opitz et al. detected an accumulation of KYN and QUIN in TDO-expressing glioblastoma cells (146), in addition to lower serum levels of TRP and KYN in glioblastoma patients. This indicates an increased transport of TRP and KYN through the blood–brain barrier. In this context it was shown that the TDO-derived KYN interfered with AhR signaling, leading to a suppression of antitumor immune responses and likewise promoted tumor-cell survival (146). Unfortunately, KYNA concentrations were not evaluated in this study. The studies of Adams et al. found no evidence for an increased production of neither the metabolites KYN, QUIN, nor KYNA in the supernatant of glioblastoma cells. However, the mRNA expression of KAT I, II, and III but not the secretion of KYNA was reduced in glioma cells compared with fetal or adult astrocytes (123), suggesting that in glioma cancer cells there is no shift to the KYNA branch compared with other cancer cells. The absence of increased KYNA production in glioma cells might be a benefit for tumor survival, since it has been shown that KYNA inhibits the proliferation and migration of human glioblastoma T98G cells (141). A shift of KYNA to QUIN production was also shown in patients with primary cervical cancer, resulting in reduced levels of serum KYNA whereas QUIN was increased concurrent with unchanged levels of TRP and KYN (122). It was assumed that the increased levels of QUIN may contribute to the restoration of energy supplies *via* formation of acetyl-CoA and NAD pathways. Decreased levels of KYNA were also detected in the urine of prostate cancer patients (25). However, whether the decrease resulted from attenuated KYNA synthesis or an impaired renal clearance was not evaluated in this study.

### Mental Disorders

It is well described that there is a link between an inflammation-induced impairment of the balance of TRP metabolism and the development of mental disorders such as depression or schizophrenia (130, 147). KYNA was found to be decreased in the blood of patients with affective psychosis (124), chronic schizophrenia (125), Alzheimer’s dementia (35, 128), cluster headache (127), and chronic migraine (126) compared with healthy subjects. Similar to the findings in glioma patients, the decrease of KYNA in blood may indicate an increased transfer of TRP or KYN through the blood–brain barrier as a substrate for local synthesis of KYNA in brain tissue. This is supported by the findings that increased levels of KYNA were detected in the CSF of patients with schizophrenia (130–132). In the CSF of patients with Alzheimer’s disease (AD), KYNA correlated with the expression of P-tau and the soluble intercellular adhesion molecule-1, which are biomarkers for inflammation (37). A link between inflammation and increased brain levels of KYNA was furthermore described in amyotrophic lateral sclerosis (ALS) patients. This study demonstrated a correlation between increased levels of KYNA in CSF with the severe clinical status of ALS (137). It is assumed that oxidative stress, glutamatergic excitotoxicity, or neuroinflammation play key roles in the pathophysiology of neurodegeneration, particularly in ALS or AD (148). Therefore, the increased production of KYNA may act as compensatory response to neurotoxic effects. The fact that KYNA was decreased in the blood of patients with a severe clinical state of ALS (137) supports the suggestion that TRP or KYN from the periphery is used as precursors for increased brain synthesis of KYNA, knowing that KYNA is hardly able to cross the blood–brain barrier (149). Increased levels of KYNA in saliva were also detected in schizophrenia patients with concurrent distress intolerance compared with distress-tolerant patients and healthy controls. This finding indicated an interference of stress with the activation of KP (129).

### Inherited Diseases/Diseases with Questionable Cause

In patients suffering from Huntington’s disease, KYNA was decreased in the CSF and several brain regions (135, 136, 150). It was suggested that this was due to a selective impairment in KYNA biosynthesis in specific brain areas of HD patients (150), resulting in an inadequate anti-inflammatory and neuroprotective response to inflammatory conditions.

Increased concentrations of KYNA in the brain were described in patients with Down syndrome (133), which exhibit similar neuropathological features as patients with AD, such as neuritic amyloid-β plaques (151). This indicates that neuro-inflammatory processes may play a role in the Down syndrome phenotype. Increased brain levels of KYNA may reflect a compensatory response to neurotoxic effects due to congenital malfunctions. Furthermore, the finding that in Down syndrome patients the urinary excretion of KYN was lower concurrent with increased excretion of KYNA, and anthranilic acid, suggests a shift of KP to the neuroprotective and antioxidative branch (134).

In conclusion, the results of the described clinical studies might indicate that the production of KYNA is a compensatory mechanism that functions to limit inflammation-induced cell and tissue damage in both brain and periphery. Furthermore, an impaired synthesis of KYNA may provoke an inadequate anti-inflammatory response characterized by, e.g., enhanced tissue damage or exceeding cell proliferation during inflammatory conditions. In tumor cells, the modulation of KYNA secretion was found to be different between the types of carcinoma. An enhanced production of KYNA by cancer cells may provoke the establishment of an immunosuppressive microenvironment for effective immune escape. Decreased levels of KYNA in the periphery of patient suffering from cerebral cancer (and also from mental disorders) might reflect an increased transfer of TRP and KYN through the blood–brain barrier. This might be a consequence of an accelerated TRP degradation due to pathological processes in brain tissue. However, also a shift to another branch of KP such as QUIN may result in the reduction of KYNA.

## KYNA, Microbiota, and Gut Homeostasis

High GPR35 expression in the gastrointestinal tract (26, 79) indicates that this receptor, and probably its ligand KYNA, could have a function in gut homeostasis (152). The potential significance of KYNA for gut health emerges from its association with various bowel diseases and colon cancer [Table 1 (30, 138, 139)], as well as the potential anti-inflammatory effects of KYNA treatment in dogs with experimental colon obstruction (71). Studies in rats and pigs have shown a high concentration of KYNA in the intestinal lumen (153, 154). The intestinal KYNA concentration increased from the proximal to the distal part of the gut, reaching ~16 μM in the distal ileum of the rat (153) and ~1.6 μM in the colon of the pig (154). The studies in rat suggest that relevant amounts of KYNA in the gut originated from the intestinal microflora, due to the relatively low concentrations in the wall of the ileum (~0.2–0.3 μM) and the food (~0.6 μM). However, certain foods and herbs may contain relatively high amounts of KYNA like broccoli (~2 μM), honey (~1 μM), basil (~74 μM), and thyme (~9 μM) (155, 156). Furthermore, the intestinal commensal *Escherichia coli* can produce and liberate KYNA through aspartate aminotransferase (AspAT) (153, 157, 158). KYNA is readily absorbed from the gut into the bloodstream (155). Interestingly, rats with the probiotic *Bifidobacteria infantis* in the gut have significantly higher KYNA levels in the blood then un-colonized control animals (159). Furthermore, blood from *B. infantis*-colonized animals exhibits a lower TNF induction after *ex vivo* challenge with LPS, which is a typical indication of an endotoxin tolerance. There are also indications that KYNA selectively regulates the growth, and thereby the composition, of the intestinal microbiota (160). In this context, the microbial-mediated KYNA catabolism, known so far from *Pseudomonas* and *Aerococcus* (60, 61), might be relevant. Interestingly, feed supplementation with very high amounts of KYNA might have a toxic/stress-inducing effect in rainbow trout (161). Hence, further studies are necessary to evaluate if a supplementation of KYNA is beneficial or detrimental to human health.

## Conclusion

Due to the proven relevance of KYNA for various diseases, it is often mooted as both a target and agent for therapeutic interventions. However, the interference of KYNA with diverse immune-related signaling pathways requires further in-depth analysis to avoid unexpected adverse consequences.

## Author Contributions

EW and JG have contributed equally to this work; they designed the manuscript and approved it for publication. AH and AR contributed to critically revising the paper.

## Conflict of Interest Statement

The authors declare that the research was conducted in the absence of any commercial or financial relationships that could be construed as a potential conflict of interest.

## References

[1] Takikawa O. Biochemical and medical aspects of the indoleamine 2, 3-dioxygenase-initiated l-tryptophan metabolism. Biochem Biophys Res Commun (2005) 338(1):12–9. 10.1016/j.bbrc.2005.09.032 16176799

[2] Wirthgen E Hoeflich A. Endotoxin-induced tryptophan degradation along the kynurenine pathway: the role of indolamine 2, 3-dioxygenase and aryl hydrocarbon receptor-mediated immunosuppressive effects in endotoxin tolerance and cancer and its implications for immunoparalysis. J Amino Acids (2015) 2015:973548. 10.1155/2015/973548 26881062 PMC4736209

[3] Arnaiz-Cot J Gonzalez J Sobrado M Baldelli P Carbone E Gandia L Allosteric modulation of α7 nicotinic receptors selectively depolarizes hippocampal interneurons, enhancing spontaneous GABAergic transmission. Eur J Neurosci (2008) 27(5):1097–110. 10.1111/j.1460-9568.2008.06077.x 18312591

[4] Kanai M Funakoshi H Takahashi H Hayakawa T Mizuno S Matsumoto K Tryptophan 2,3-dioxygenase is a key modulator of physiological neurogenesis and anxiety-related behavior in mice. Mol Brain (2009) 2:8. 10.1186/1756-6606-2-8 19323847 PMC2673217

[5] Le Floc’h N Otten W Merlot E. Tryptophan metabolism, from nutrition to potential therapeutic applications. Amino Acids (2011) 41(5):1195–205. 10.1007/s00726-010-0752-7 20872026

[6] Isenovic E Zakula Z Koricanac G Ribarac-Stepic N. Comparative analysis of tryptophan oxygenase activity and glucocorticoid receptor under the influence of insulin. Physiol Res (2008) 57(1):101. 17223727 10.33549/physiolres.931135

[7] Niimi S Nakamura T Nawa K Ichihara A. Hormonal regulation of translatable mRNA of tryptophan 2, 3-dioxygenase in primary cultures of adult rat hepatocytes. J Biochem (1983) 94(5):1697–706. 6361014

[8] Schubart UK Regulation of gene expression in rat hepatocytes and hepatoma cells by insulin: quantitation of messenger ribonucleic acid’s coding for tyrosine aminotransferase, tryptophan oxygenase, and phosphoenolpyruvate carboxykinase. Endocrinology (1986) 119(4):1741–9. 10.1210/endo-119-4-1741 2875868

[9] Mándi Y Vécsei L The kynurenine system and immunoregulation. J Neural Transm (2012) 119(2):197–209. 10.1007/s00702-011-0681-y 21744051

[10] Liu X Newton RC Friedman SM Scherle PA. Indoleamine 2, 3-dioxygenase, an emerging target for anti-cancer therapy. Curr Cancer Drug Targets (2009) 9(8):938–52. 10.2174/156800909790192374 20025603

[11] Stone TW Darlington LG. Endogenous kynurenines as targets for drug discovery and development. Nat Rev Drug Discov (2002) 1(8):609–20. 10.1038/nrd870 12402501

[12] Metz R DuHadaway JB Kamasani U Laury-Kleintop L Muller AJ Prendergast GC. Novel tryptophan catabolic enzyme IDO2 is the preferred biochemical target of the antitumor indoleamine 2, 3-dioxygenase inhibitory compound d-1-methyl-tryptophan. Cancer Res (2007) 67(15):7082–7. 10.1158/0008-5472.CAN-07-1872 17671174

[13] Yoshida R Imanishi J Oku T Kishida T Hayaishi O. Induction of pulmonary indoleamine 2, 3-dioxygenase by interferon. Proc Natl Acad Sci U S A (1981) 78(1):129–32. 10.1073/pnas.78.1.129 6165986 PMC319004

[14] Werner ER Bitterlich G Fuchs D Hausen A Reibnegger G Szabo G Human macrophages degrade tryptophan upon induction by interferon-gamma. Life Sci (1987) 41(3):273–80. 10.1016/0024-3205(87)90149-4 3110526

[15] Ruddick JP Evans AK Nutt DJ Lightman SL Rook GA Lowry CA. Tryptophan metabolism in the central nervous system: medical implications. Expert Rev Mol Med (2006) 8(20):1–27. 10.1017/S1462399406000068 16942634

[16] Anderson G Maes M. Interactions of tryptophan and its catabolites with melatonin and the alpha 7 nicotinic receptor in central nervous system and psychiatric disorders: role of the aryl hydrocarbon receptor and direct mitochondria regulation. Int J Tryptophan Res (2017) 10:1178646917691738. 10.1177/1178646917691738 28469467 PMC5398327

[17] Majláth Z Török N Toldi J Vécsei L. Memantine and kynurenic acid: current neuropharmacological aspects. Curr Neuropharmacol (2016) 14(2):200–9. 10.2174/1570159X14666151113123221 26564141 PMC4825950

[18] Hilmas C Pereira EF Alkondon M Rassoulpour A Schwarcz R Albuquerque EX The brain metabolite kynurenic acid inhibits α7 nicotinic receptor activity and increases non-α7 nicotinic receptor expression: physiopathological implications. J Neurosci (2001) 21(19):7463–73. 11567036 10.1523/JNEUROSCI.21-19-07463.2001 PMC6762893

[19] Stone TW. Kynurenic acid blocks nicotinic synaptic transmission to hippocampal interneurons in young rats. Eur J Neurosci (2007) 25(9):2656–65. 10.1111/j.1460-9568.2007.05540.x 17459105

[20] Mok MS Fricker A-C Weil A Kew JN. Electrophysiological characterisation of the actions of kynurenic acid at ligand-gated ion channels. Neuropharmacology (2009) 57(3):242–9. 10.1016/j.neuropharm.2009.06.003 19523966

[21] Dobelis P Staley KJ Cooper DC. Lack of modulation of nicotinic acetylcholine alpha-7 receptor currents by kynurenic acid in adult hippocampal interneurons. PLoS One (2012) 7(7):e41108. 10.1371/journal.pone.0041108 22848433 PMC3405093

[22] Moroni F Cozzi A Sili M Mannaioni G. Kynurenic acid: a metabolite with multiple actions and multiple targets in brain and periphery. J Neural Transm (2012) 119(2):133–9. 10.1007/s00702-011-0763-x 22215208

[23] Forrest CM Gould SR Darlington LG Stone TW Levels of purine, kynurenine and lipid peroxidation products in patients with inflammatory bowel disease. Adv Exp Med Biol (2003) 527:395–400. 10.1007/978-1-4615-0135-0_46 15206756

[24] Dabrowski W Kocki T Pilat J Parada-Turska J Malbrain MLNG. Changes in plasma kynurenic acid concentration in septic shock patients undergoing continuous veno-venous haemofiltration. Inflammation (2014) 37(1):223–34. 10.1007/s10753-013-9733-9 24043287 PMC3929023

[25] Gkotsos G Virgiliou C Lagoudaki I Sardeli C Raikos N Theodoridis G The role of sarcosine, uracil, and kynurenic acid metabolism in urine for diagnosis and progression monitoring of prostate cancer. Metabolites (2017) 7(1):9 10.3390/metabo7010009 PMC5372212 28241496

[26] Wang J Simonavicius N Wu X Swaminath G Reagan J Tian H Kynurenic acid as a ligand for orphan G protein-coupled receptor GPR35. J Biol Chem (2006) 281(31):22021–8. 10.1074/jbc.M603503200 16754668

[27] DiNatale BC Murray IA Schroeder JC Flaveny CA Lahoti TS Laurenzana EM Kynurenic acid is a potent endogenous aryl hydrocarbon receptor ligand that synergistically induces interleukin-6 in the presence of inflammatory signaling. Toxicol Sci (2010) 115(1):89–97. 10.1093/toxsci/kfq024 20106948 PMC2855350

[28] Stazka J Luchowski P Wielosz M Kleinrok Z Urbanska EM. Endothelium-dependent production and liberation of kynurenic acid by rat aortic rings exposed to L-kynurenine. Eur J Pharmacol (2002) 448(2–3):133–7. 10.1016/S0014-2999(02)01943-X 12144932

[29] Matysik-Wozniak A Junemann A Turski WA Wnorowski A Jozwiak K Paduch R The presence of kynurenine aminotransferases in the human cornea: evidence from bioinformatics analysis of gene expression and immunohistochemical staining. Mol Vis (2017) 23:364–71. 28706436 PMC5501688

[30] Walczak K Dabrowski W Langner E Zgrajka W Pilat J Kocki T Kynurenic acid synthesis and kynurenine aminotransferases expression in colon derived normal and cancer cells. Scand J Gastroenterol (2011) 46(7–8):903–12. 10.3109/00365521.2011.579159 21615226

[31] Asp L Johansson AS Mann A Owe-Larsson B Urbanska EM Kocki T Effects of pro-inflammatory cytokines on expression of kynurenine pathway enzymes in human dermal fibroblasts. J Inflamm (2011) 8:25. 10.1186/1476-9255-8-25 21982155 PMC3204223

[32] Liu JJ Raynal S Bailbe D Gausseres B Carbonne C Autier V Expression of the kynurenine pathway enzymes in the pancreatic islet cells. Activation by cytokines and glucolipotoxicity. Biochim Biophys Acta (2015) 1852(5):980–91. 10.1016/j.bbadis.2015.02.001 25675848

[33] Jones SP Franco NF Varney B Sundaram G Brown DA de Bie J Expression of the kynurenine pathway in human peripheral blood mononuclear cells: implications for inflammatory and neurodegenerative disease. PLoS One (2015) 10(6):e0131389. 10.1371/journal.pone.0131389 26114426 PMC4482723

[34] Agudelo LZ Femenia T Orhan F Porsmyr-Palmertz M Goiny M Martinez-Redondo V Skeletal muscle PGC-1alpha1 modulates kynurenine metabolism and mediates resilience to stress-induced depression. Cell (2014) 159(1):33–45. 10.1016/j.cell.2014.07.051 25259918

[35] Hartai Z Juhasz A Rimanoczy A Janaky T Donko T Dux L Decreased serum and red blood cell kynurenic acid levels in Alzheimer’s disease. Neurochem Int (2007) 50(2):308–13. 10.1016/j.neuint.2006.08.012 17023091

[36] Baran H Amann G Lubec B Lubec G Kynurenic acid and kynurenine aminotransferase in heart. Pediatr Res (1997) 41(3):404–10. 10.1203/00006450-199703000-00017 9078543

[37] Wennström M Nielsen HM Orhan F Londos E Minthon L Erhardt S Kynurenic acid levels in cerebrospinal fluid from patients with Alzheimer’s disease or dementia with Lewy bodies. Int J Tryptophan Res (2014) 7:1–7. 10.4137/ijtr.s13958 24855376 PMC4011721

[38] Okuno E Nakamura M Schwarcz R Two kynurenine aminotransferases in human brain. Brain Res (1991) 542:307–12. 10.1016/0006-8993(91)91583-m 2029638

[39] Guidetti P Okuno E Schwarcz R Characterization of rat brain kynurenine aminotransferases I and II. J Neurosci Res (1997) 50:457–65. 10.1002/(SICI)1097-4547(19971101)50:3<457::AID-JNR12>3.0.CO;2-3 9364331

[40] Schwarcz R Pellicciari R Manipulation of brain kynurenines: glial targets, neuronal effects, and clinical opportunities. J Pharmacol Exp Ther (2002) 303:1–10. 10.1124/jpet.102.034439 12235226

[41] Han Q Li J Li J pH dependence, substrate specificity and inhibition of human kynurenine aminotransferase I. Eur J Biochem (2004) 271:4804–14. 10.1111/j.1432-1033.2004.04446.x 15606768

[42] Yu P Li Z Zhang L Tagle DA Cai T Characterization of kynurenine aminotransferase III, a novel member of a phylogenetically conserved KAT family. Gene (2006) 365:111–8. 10.1016/j.gene.2005.09.034 16376499

[43] Han Q Robinson H Cai T Tagle DA Li J. Biochemical and structural properties of mouse kynurenine aminotransferase III. Mol Cell Biol (2009) 29:784–93. 10.1128/mcb.01272-08 19029248 PMC2630683

[44] Noguchi T Minatogawa Y Okuno E Nakatani M Morimoto M Kido R Purification and characterization of kynurenine-2-oxoglutarate aminotransferase from the liver, brain and small intestine of rats. Biochem J (1975) 151:399–406. 10.1042/bj1510399 1218085 PMC1172370

[45] Baran H Schwarcz R Regional differences in the ontogenetic pattern of kynurenine aminotransferase in the rat brain. Brain Res Dev Brain Res (1993) 74:283–6. 10.1016/0165-3806(93)90014-2 8403387

[46] Guillemin GJ Kerr SJ Smythe GA Smith DG Kapoor V Armati PJ Kynurenine pathway metabolism in human astrocytes: a paradox for neuronal protection. J Neurochem (2001) 78(4):842–53. 10.1046/j.1471-4159.2001.00498.x 11520905

[47] Guillemin GJ Cullen KM Lim CK Smythe GA Garner B Kapoor V Characterization of the kynurenine pathway in human neurons. J Neurosci (2007) 27(47):12884–92. 10.1523/jneurosci.4101-07.2007 18032661 PMC6673280

[48] Parrott JM Redus L O’Connor JC. Kynurenine metabolic balance is disrupted in the hippocampus following peripheral lipopolysaccharide challenge. J Neuroinflammation (2016) 13:124. 10.1186/s12974-016-0590-y 27233247 PMC4884395

[49] Blanco Ayala T Lugo Huitrón R Carmona Aparicio L Ramírez Ortega D González Esquivel D Pedraza Chaverrí J Alternative kynurenic acid synthesis routes studied in the rat cerebellum. Front Cell Neurosci (2015) 9:178. 10.3389/fncel.2015.00178 26041992 PMC4435238

[50] Hardeland R Melatonin and other tryptophan metabolites: rhythms outside the animal world and some novel, presumably universal pathways. In: Fanjul-Moles ML, editor. Comparative aspects of circadian rhythms, Kerala, India: Transworld Research Network (2008). p. 1–17.

[51] Zsizsik BK Hardeland R. Formation of kynurenic and xanthurenic acids from kynurenine and 3-hydroxykynurenine in the dinoflagellate *Lingulodinium polyedrum*: role of a novel, oxidative pathway. Comp Biochem Physiol C Toxicol Pharmacol (2002) 133(3):383–92. 10.1016/S1532-0456(02)00126-6 12379423

[52] Politi V Lavaggi MV Di Stazio G Margonelli A Indole-3-pyruvic acid as a direct precursor of kynurenic acid. Adv Exp Med Biol (1991) 294:515–8. 10.1007/978-1-4684-5952-4_57 1772085

[53] Zsizsik B Hardeland R A novel pathway of kynurenic acid formation: oxidation of l-kynurenine by H_2_O_2_ in the presence and absence of peroxidase. In: Hardeland R, editor. Actions and Redox Properties of Melatonin and Other Aromatic Amino Acid Metabolites: Reports from the Laboratories of Metabolism Research and Chronobiology at the Institute of Zoology and Anthropology, University of Göttingen, Germany. Göttingen: Cuvillier (2001). p. 168–76.

[54] Wirthgen E Tuchscherer M Otten W Domanska G Wollenhaupt K Tuchscherer A Activation of indoleamine 2, 3-dioxygenase by LPS in a porcine model. Innate Immun (2014) 20(1):30–9. 10.1177/1753425913481252 23606516

[55] Kiank C Zeden J-P Drude S Domanska G Fusch G Otten W Psychological stress-induced, IDO1-dependent tryptophan catabolism: implications on immunosuppression in mice and humans. PLoS One (2010) 5(7):e11825. 10.1371/journal.pone.0011825 20689575 PMC2911374

[56] Murachi T Tsukada K Hayaishi O Metabolic fate of kynurenic acid-C-14 intraperitoneally administered to animals. Biochemistry (1963) 2:304–8. 10.1021/bi00902a021 13936686

[57] Vécsei L Beal MF Intracerebroventricular injection of kynurenic acid, but not kynurenine, induces ataxia and stereotyped behavior in rats. Brain Res Bull (1990) 25(4):623–7. 10.1016/0361-9230(90)90123-H 2271966

[58] Kaihara M Price JM The metabolism of quinaldic acid, kynurenic acid, and xanthurenic acid in the rabbit. J Biol Chem (1962) 237:1727–9. 14453131

[59] Kaihara M Price JM Takahashi H The conversion of kynurenic acid to quinaldic acid by humans and rats. J Biol Chem (1956) 223(2):705–8. 13385219

[60] Hayaishi O Taniuchi H Tashiro M Kuno S Studies on the metabolism of kynurenic acid. I. The formation of l-glutamic acid, d- and l-alanine, and acetic acid from kynurenic acid by *Pseudomonas* extracts. J Biol Chem (1961) 236:2492–7. 13712440

[61] Dagley S Johnson PA Microbial oxidation of kynurenic, xanthurenic and picolinic acids. Biochim Biophys Acta (1963) 78(4):577–87. 10.1016/0006-3002(63)91023-0 14089438

[62] Malaczewska J Siwicki AK Wojcik RM Turski WA Kaczorek E. The effect of kynurenic acid on the synthesis of selected cytokines by murine splenocytes – in vitro and ex vivo studies. Cent Eur J Immunol (2016) 41(1):39–46. 10.5114/ceji.2016.58815 27095921 PMC4829820

[63] Barth MC Ahluwalia N Anderson TJT Hardy GJ Sinha S Alvarez-Cardona JA Kynurenic acid triggers firm arrest of leukocytes to vascular endothelium under flow conditions. J Biol Chem (2009) 284(29):19189–95. 10.1074/jbc.M109.024042 19473985 PMC2740542

[64] Maaetoft-Udsen K Shimoda LM Frokiaer H Turner H. Aryl hydrocarbon receptor ligand effects in RBL2H3 cells. J Immunotoxicol (2012) 9(3):327–37. 10.3109/1547691x.2012.661802 22471748 PMC3529589

[65] Steiner L Gold M Mengel D Dodel R Bach J-P The endogenous α7 nicotinic acetylcholine receptor antagonist kynurenic acid modulates amyloid-β-induced inflammation in BV-2 microglial cells. J Neurol Sci (2014) 344(1):94–9. 10.1016/j.jns.2014.06.032 25064444

[66] Tiszlavicz Z Németh B Fülöp F Vécsei L Tápai K Ocsovszky I Different inhibitory effects of kynurenic acid and a novel kynurenic acid analogue on tumour necrosis factor-α (TNF-α) production by mononuclear cells, HMGB1 production by monocytes and HNP1-3 secretion by neutrophils. Naunyn Schmiedebergs Arch Pharmacol (2011) 383(5):447–55. 10.1007/s00210-011-0605-2 21336543

[67] Moroni F Fossati S Chiarugi A Cozzi A Kynurenic acid actions in brain and periphery. Int Congr Ser (2007) 1304:305–13. 10.1016/j.ics.2007.07.016

[68] Fallarini S Magliulo L Paoletti T de Lalla C Lombardi G. Expression of functional GPR35 in human iNKT cells. Biochem Biophys Res Commun (2010) 398(3):420–5. 10.1016/j.bbrc.2010.06.091 20599711

[69] Salimi Elizei S Poormasjedi-Meibod M-S Wang X Kheirandish M Ghahary A. Kynurenic acid downregulates IL-17/1L-23 axis in vitro. Mol Cell Biochem (2017) 431(1–2):55–65. 10.1007/s11010-017-2975-3 28285360

[70] Gaffen SL Jain R Garg AV Cua DJ. The IL-23-IL-17 immune axis: from mechanisms to therapeutic testing. Nat Rev Immunol (2014) 14(9):585–600. 10.1038/nri3707 25145755 PMC4281037

[71] Kaszaki J Palásthy Z érczes D Rácz A Torday C Varga G Kynurenic acid inhibits intestinal hypermotility and xanthine oxidase activity during experimental colon obstruction in dogs. Neurogastroenterol Motil (2008) 20(1):53–62. 10.1111/j.1365-2982.2007.00989.x 17973632

[72] Platten M von Knebel Doeberitz N Oezen I Wick W Ochs K. Cancer immunotherapy by targeting IDO1/TDO and their downstream effectors. Front Immunol (2015) 5:673. 10.3389/fimmu.2014.00673 25628622 PMC4290671

[73] Lugo-Huitrón R Blanco-Ayala T Ugalde-Muñiz P Carrillo-Mora P Pedraza-Chaverrí J Silva-Adaya D On the antioxidant properties of kynurenic acid: free radical scavenging activity and inhibition of oxidative stress. Neurotoxicol Teratol (2011) 33(5):538–47. 10.1016/j.ntt.2011.07.002 21763768

[74] Perez-Gonzalez A Alvarez-Idaboy JR Galano A. Free-radical scavenging by tryptophan and its metabolites through electron transfer based processes. J Mol Model (2015) 21:213. 10.1007/s00894-015-2758-2 26224603

[75] Shajib MS Khan WI. The role of serotonin and its receptors in activation of immune responses and inflammation. Acta Physiol (Oxf) (2015) 213(3):561–74. 10.1111/apha.12430 25439045

[76] Reiter RJ Calvo JR Karbownik M Qi W Tan DX Melatonin and its relation to the immune system and inflammation. Ann N Y Acad Sci (2000) 917:376–86. 10.1111/j.1749-6632.2000.tb05402.x 11268363

[77] Skene DJ Papagiannidou E Hashemi E Snelling J Lewis DF Fernandez M Contribution of CYP1A2 in the hepatic metabolism of melatonin: studies with isolated microsomal preparations and liver slices. J Pineal Res (2001) 31(4):333–42. 10.1034/j.1600-079X.2001.310408.x 11703563

[78] Yang Y Lu JYL Wu X Summer S Whoriskey J Saris C G-protein-coupled receptor 35 is a target of the asthma drugs cromolyn disodium and nedocromil sodium. Pharmacology (2010) 86(1):1–5. 10.1159/000314164 20559017

[79] Taniguchi Y Tonai-Kachi H Shinjo K. Zaprinast, a well-known cyclic guanosine monophosphate-specific phosphodiesterase inhibitor, is an agonist for GPR35. FEBS Lett (2006) 580(21):5003–8. 10.1016/j.febslet.2006.08.015 16934253

[80] Maravillas-Montero JL Burkhardt AM Hevezi PA Carnevale CD Smit MJ Zlotnik A. Cutting edge: GPR35/CXCR8 is the receptor of the mucosal chemokine CXCL17. J Immunol (2015) 194(1):29–33. 10.4049/jimmunol.1401704 25411203 PMC4355404

[81] Guo J Williams DJ Puhl HL Ikeda SR. Inhibition of N-type calcium channels by activation of GPR35, an orphan receptor, heterologously expressed in rat sympathetic neurons. J Pharmacol Exp Ther (2008) 324(1):342–51. 10.1124/jpet.107.127266 17940199

[82] Berlinguer-Palmini R Masi A Narducci R Cavone L Maratea D Cozzi A GPR35 activation reduces Ca^2+^ transients and contributes to the kynurenic acid-dependent reduction of synaptic activity at CA3-CA1 synapses. PLoS One (2013) 8(11):e82180. 10.1371/journal.pone.0082180 24312407 PMC3843712

[83] Jenkins L Brea J Smith Nicola J Hudson Brian D Reilly G Bryant Nia J Identification of novel species-selective agonists of the G-protein-coupled receptor GPR35 that promote recruitment of β-arrestin-2 and activate Gα13. Biochem J (2010) 432(3):451–9. 10.1042/bj20101287 20919992

[84] Walczak K Turski WA Rajtar G. Kynurenic acid inhibits colon cancer proliferation in vitro: effects on signaling pathways. Amino Acids (2014) 46(10):2393–401. 10.1007/s00726-014-1790-3 25012123 PMC4168223

[85] Goldsmith ZG Dhanasekaran DNG Protein regulation of MAPK networks. Oncogene (2007) 26(22):3122–42. 10.1038/sj.onc.1210407 17496911

[86] Shevtsov SP Haq S Force T Activation of β-catenin signaling pathways by classical G-protein-coupled receptors: mechanisms and consequences in cycling and non-cycling cells. Cell Cycle (2006) 5(20):2295–300. 10.4161/cc.5.20.3357 17035736

[87] Han C Chen T Yang M Li N Liu H Cao X. Human SCAMP5, a novel secretory carrier membrane protein, facilitates calcium-triggered cytokine secretion by interaction with SNARE machinery. J Immunol (2009) 182(5):2986–96. 10.4049/jimmunol.0802002 19234194

[88] Murray RZ Stow JL. Cytokine secretion in macrophages: SNAREs, Rabs, and membrane trafficking. Front Immunol (2014) 5:538. 10.3389/fimmu.2014.00538 25386181 PMC4209870

[89] Yang IH Wong J-H Chang C-M Chen B-K Tsai Y-T Chen W-C Involvement of intracellular calcium mobilization in IL-8 activation in human retinal pigment epithelial cells. Invest Ophthalmol Vis Sci (2015) 56(2):761–9. 10.1167/iovs.14-15299 25593029

[90] Hoesel B Schmid JA The complexity of NF-κB signaling in inflammation and cancer. Mol Cancer (2013) 12(1):86 10.1186/1476-4598-12-86 23915189 PMC3750319

[91] Raker VK Becker C Steinbrink K. The cAMP pathway as therapeutic target in autoimmune and inflammatory diseases. Front Immunol (2016) 7:123. 10.3389/fimmu.2016.00123 27065076 PMC4814577

[92] Wehbi VL Taskén K Molecular mechanisms for cAMP-mediated immunoregulation in T cells – role of anchored protein kinase A signaling units. Front Immunol (2016) 7:222 10.3389/fimmu.2016.00222 27375620 PMC4896925

[93] Zhang Y Wang X Yang H Liu H Lu Y Han L Kinase AKT controls innate immune cell development and function. Immunology (2013) 140(2):143–52. 10.1111/imm.12123 23692658 PMC3784161

[94] Arthur JSC Ley SC. Mitogen-activated protein kinases in innate immunity. Nat Rev Immunol (2013) 13(9):679–92. 10.1038/nri3495 23954936

[95] Silva-García O Valdez-Alarcón JJ Baizabal-Aguirre VM The Wnt/β-catenin signaling pathway controls the inflammatory response in infections caused by pathogenic bacteria. Mediators Inflamm (2014) 2014:7 10.1155/2014/310183 PMC4127235 25136145

[96] Nguyen NT Nakahama T Le DH Van Son L Chu HH Kishimoto T. Aryl hydrocarbon receptor and kynurenine: recent advances in autoimmune disease research. Front Immunol (2014) 5:551. 10.3389/fimmu.2014.00551 25400638 PMC4212680

[97] Sekine H Mimura J Oshima M Okawa H Kanno J Igarashi K Hypersensitivity of aryl hydrocarbon receptor-deficient mice to lipopolysaccharide-induced septic shock. Mol Cell Biol (2009) 29(24):6391–400. 10.1128/MCB.00337-09 19822660 PMC2786870

[98] Thatcher TH Maggirwar SB Baglole CJ Lakatos HF Gasiewicz TA Phipps RP Aryl hydrocarbon receptor-deficient mice develop heightened inflammatory responses to cigarette smoke and endotoxin associated with rapid loss of the nuclear factor-kappaB component RelB. Am J Pathol (2007) 170(3):855–64. 10.2353/ajpath.2007.060391 17322371 PMC1864867

[99] Thatcher TH Williams MA Pollock SJ McCarthy CE Lacy SH Phipps RP Endogenous ligands of the aryl hydrocarbon receptor regulate lung dendritic cell function. Immunology (2016) 147(1):41–54. 10.1111/imm.12540 26555456 PMC4693882

[100] Baglole CJ Maggirwar SB Gasiewicz TA Thatcher TH Phipps RP Sime PJ The aryl hydrocarbon receptor attenuates tobacco smoke-induced cyclooxygenase-2 and prostaglandin production in lung fibroblasts through regulation of the NF-κB family member RelB. J Biol Chem (2008) 283(43):28944–57. 10.1074/jbc.M800685200 18697742 PMC2570856

[101] Zhang S Patel A Chu C Jiang W Wang L Welty SE Aryl hydrocarbon receptor is necessary to protect fetal human pulmonary microvascular endothelial cells against hyperoxic injury: mechanistic roles of antioxidant enzymes and RelB. Toxicol Appl Pharmacol (2015) 286(2):92–101. 10.1016/j.taap.2015.03.023 25831079 PMC4458211

[102] García-Lara L Pérez-Severiano F González-Esquivel D Elizondo G Segovia J. Absence of aryl hydrocarbon receptors increases endogenous kynurenic acid levels and protects mouse brain against excitotoxic insult and oxidative stress. J Neurosci Res (2015) 93(9):1423–33. 10.1002/jnr.23595 26013807

[103] Quintana FJ Sherr DH. Aryl hydrocarbon receptor control of adaptive immunity. Pharmacol Rev (2013) 65(4):1148–61. 10.1124/pr.113.007823 23908379 PMC3799235

[104] Bessede A Gargaro M Pallotta MT Matino D Servillo G Brunacci C Aryl hydrocarbon receptor control of a disease tolerance defence pathway. Nature (2014) 511(7508):184–90. 10.1038/nature13323 24930766 PMC4098076

[105] Zeden J Fusch G Holtfreter B Schefold J Reinke P Domanska G Excessive tryptophan catabolism along the kynurenine pathway precedes ongoing sepsis in critically ill patients. Anaesth Intensive Care (2010) 38(2):307. 20369765 10.1177/0310057X1003800213

[106] Lee KJ Jung KH Cho JY Lee ST Kim HS Shim JH High-fat diet and voluntary chronic aerobic exercise recover altered levels of aging-related tryptophan metabolites along the kynurenine pathway. Exp Neurobiol (2017) 26(3):132–40. 10.5607/en.2017.26.3.132 28680298 PMC5491581

[107] Vogel CFA Sciullo E Li W Wong P Lazennec G Matsumura F. RelB, a new partner of aryl hydrocarbon receptor-mediated transcription. Mol Endocrinol (2007) 21(12):2941–55. 10.1210/me.2007-0211 17823304 PMC2346533

[108] Tanaka T Narazaki M Kishimoto T. IL-6 in inflammation, immunity, and disease. Cold Spring Harb Perspect Biol (2014) 6(10):a016295. 10.1101/cshperspect.a016295 25190079 PMC4176007

[109] Hegde S Pahne J Smola-Hess S Novel immunosuppressive properties of interleukin-6 in dendritic cells: inhibition of NF-κB binding activity and CCR7 expression. FASEB J (2004) 18(12):1439–41. 10.1096/fj.03-0969fje 15247147

[110] Scheller J Chalaris A Schmidt-Arras D Rose-John S. The pro- and anti-inflammatory properties of the cytokine interleukin-6. Biochim Biophys Acta (2011) 1813(5):878–88. 10.1016/j.bbamcr.2011.01.034 21296109

[111] Fisher DT Appenheimer MM Evans SS. The two faces of IL-6 in the tumor microenvironment. Semin Immunol (2014) 26(1):38–47. 10.1016/j.smim.2014.01.008 24602448 PMC3970580

[112] Litzenburger UM Opitz CA Sahm F Rauschenbach KJ Trump S Winter M Constitutive IDO expression in human cancer is sustained by an autocrine signaling loop involving IL-6, STAT3 and the AHR. Oncotarget (2014) 5(4):1038. 10.18632/oncotarget.1637 24657910 PMC4011581

[113] Murray IA Patterson AD Perdew GH Ah receptor ligands in cancer: friend and foe. Nat Rev Cancer (2014) 14(12):801–14. 10.1038/nrc3846 25568920 PMC4401080

[114] Hartai Z Klivenyi P Janaky T Penke B Dux L Vecsei L. Kynurenine metabolism in multiple sclerosis. Acta Neurol Scand (2005) 112(2):93–6. 10.1111/j.1600-0404.2005.00442.x 16008534

[115] Ristagno G Latini R Vaahersalo J Masson S Kurola J Varpula T Early activation of the kynurenine pathway predicts early death and long-term outcome in patients resuscitated from out-of-hospital cardiac arrest. J Am Heart Assoc (2014) 3(4):e001094 10.1161/jaha.114.001094 25092787 PMC4310405

[116] Parada-Turska J Zgrajka W Majdan M. Kynurenic acid in synovial fluid and serum of patients with rheumatoid arthritis, spondyloarthropathy, and osteoarthritis. J Rheumatol (2013) 40(6):903–9. 10.3899/jrheum.121035 23588943

[117] Igari T Tsuchizawa M Shimamura T. Alteration of tryptophan metabolism in the synovial fluid of patients with rheumatoid arthritis and osteoarthritis. Tohoku J Exp Med (1987) 153(2):79–86. 10.1620/tjem.153.79 3500530

[118] Oxenkrug GF Increased plasma levels of xanthurenic and kynurenic acids in type 2 diabetes. Mol Neurobiol (2015) 52(2):805–10. 10.1007/s12035-015-9232-0 26055228 PMC4558247

[119] Schefold JC Zeden JP Fotopoulou C von Haehling S Pschowski R Hasper D Increased indoleamine 2,3-dioxygenase (IDO) activity and elevated serum levels of tryptophan catabolites in patients with chronic kidney disease: a possible link between chronic inflammation and uraemic symptoms. Nephrol Dial Transplant (2009) 24(6):1901–8. 10.1093/ndt/gfn739 19155537

[120] Kuc D Rahnama M Tomaszewski T Rzeski W Wejksza K Urbanik-Sypniewska T Kynurenic acid in human saliva – does it influence oral microflora? Pharmacol Rep (2006) 58(3):393–8. 16845213

[121] Sagan D Kocki T Patel S Kocki J. Utility of kynurenic acid for non-invasive detection of metastatic spread to lymph nodes in non-small cell lung cancer. Int J Med Sci (2015) 12(2):146–53. 10.7150/ijms.7541 25589891 PMC4293180

[122] Fotopoulou C Sehouli J Pschowski R von Haehling S Domanska G Braicu EI Systemic changes of tryptophan catabolites via the indoleamine-2,3-dioxygenase pathway in primary cervical cancer. Anticancer Res (2011) 31(8):2629–35. 21778315

[123] Adams S Teo C McDonald KL Zinger A Bustamante S Lim CK Involvement of the kynurenine pathway in human glioma pathophysiology. PLoS One (2014) 9(11):e112945. 10.1371/journal.pone.0112945 25415278 PMC4240539

[124] Wurfel BE Drevets WC Bliss SA McMillin JR Suzuki H Ford BN Serum kynurenic acid is reduced in affective psychosis. Transl Psychiatry (2017) 7(5):e1115. 10.1038/tp.2017.88 28463241 PMC5534956

[125] Szymona K Zdzisinska B Karakula-Juchnowicz H Kocki T Kandefer-Szerszen M Flis M Correlations of kynurenic acid, 3-hydroxykynurenine, sIL-2R, IFN-alpha, and IL-4 with clinical symptoms during acute relapse of schizophrenia. Neurotox Res (2017) 32(1):17–26. 10.1007/s12640-017-9714-0 28275903

[126] Curto M Lionetto L Negro A Capi M Fazio F Giamberardino MA Altered kynurenine pathway metabolites in serum of chronic migraine patients. J Headache Pain (2015) 17:47. 10.1186/s10194-016-0638-5 27130315 PMC4851673

[127] Curto M Lionetto L Negro A Capi M Perugino F Fazio F Altered serum levels of kynurenine metabolites in patients affected by cluster headache. J Headache Pain (2015) 17:27. 10.1186/s10194-016-0620-2 27000870 PMC4801826

[128] Gulaj E Pawlak K Bien B Pawlak D. Kynurenine and its metabolites in Alzheimer’s disease patients. Adv Med Sci (2010) 55(2):204–11. 10.2478/v10039-010-0023-6 20639188

[129] Chiappelli J Pocivavsek A Nugent KL Notarangelo FM Kochunov P Rowland LM Stress-induced increase in kynurenic acid as a potential biomarker for patients with schizophrenia and distress intolerance. JAMA Psychiatry (2014) 71(7):761–8. 10.1001/jamapsychiatry.2014.243 24806441 PMC4219570

[130] Erhardt S Schwieler L Nilsson L Linderholm K Engberg G. The kynurenic acid hypothesis of schizophrenia. Physiol Behav (2007) 92(1):203–9. 10.1016/j.physbeh.2007.05.025 17573079

[131] Linderholm KR Skogh E Olsson SK Dahl ML Holtze M Engberg G Increased levels of kynurenine and kynurenic acid in the CSF of patients with schizophrenia. Schizophr Bull (2012) 38(3):426–32. 10.1093/schbul/sbq086 20729465 PMC3329991

[132] Heyes MP Lackner A. Increased cerebrospinal fluid quinolinic acid, kynurenic acid, and l-kynurenine in acute septicemia. J Neurochem (1990) 55(1):338–41. 10.1111/j.1471-4159.1990.tb08857.x 2141357

[133] Baran H Cairns N Lubec B Lubec G. Increased kynurenic acid levels and decreased brain kynurenine aminotransferase I in patients with Down syndrome. Life Sci (1996) 58(21):1891–9. 10.1016/0024-3205(96)00173-7 8637415

[134] Uberos J Romero J Molina-Carballo A Munoz-Hoyos A. Melatonin and elimination of kynurenines in children with Down’s syndrome. J Pediatr Endocrinol Metab (2010) 23(3):277–82. 10.1515/JPEM.2010.23.3.277 20480727

[135] Beal MF Matson WR Storey E Milbury P Ryan EA Ogawa T Kynurenic acid concentrations are reduced in Huntington’s disease cerebral cortex. J Neurol Sci (1992) 108(1):80–7. 10.1016/0022-510X(92)90191-M 1385624

[136] Beal MF Matson WR Swartz KJ Gamache PH Bird ED. Kynurenine pathway measurements in Huntington’s disease striatum: evidence for reduced formation of kynurenic acid. J Neurochem (1990) 55(4):1327–39. 10.1111/j.1471-4159.1990.tb03143.x 2144582

[137] Ilzecka J Kocki T Stelmasiak Z Turski WA. Endogenous protectant kynurenic acid in amyotrophic lateral sclerosis. Acta Neurol Scand (2003) 107(6):412–8. 10.1034/j.1600-0404.2003.00076.x 12757473

[138] Clarke G Fitzgerald P Cryan JF Cassidy EM Quigley EM Dinan TG. Tryptophan degradation in irritable bowel syndrome: evidence of indoleamine 2,3-dioxygenase activation in a male cohort. BMC Gastroenterol (2009) 9(1):6. 10.1186/1471-230x-9-6 19154614 PMC2648992

[139] Christmas DM Badawy AAB Hince D Davies SJC Probert C Creed T Increased serum free tryptophan in patients with diarrhea-predominant irritable bowel syndrome. Nutr Res (2010) 30(10):678–88. 10.1016/j.nutres.2010.09.009 21056283

[140] Walczak K Zurawska M Kis J Starownik R Zgrajka W Bar K Kynurenic acid in human renal cell carcinoma: its antiproliferative and antimigrative action on Caki-2 cells. Amino Acids (2012) 43(4):1663–70. 10.1007/s00726-012-1247-5 22349835

[141] Walczak K Deneka-Hannemann S Jarosz B Zgrajka W Stoma F Trojanowski T Kynurenic acid inhibits proliferation and migration of human glioblastoma T98G cells. Pharmacol Rep (2014) 66(1):130–6. 10.1016/j.pharep.2013.06.007 24905318

[142] Walczak K Turski WA Rzeski W. Kynurenic acid enhances expression of p21 Waf1/Cip1 in colon cancer HT-29 cells. Pharmacol Rep (2012) 64(3):745–50. 10.1016/S1734-1140(12)70870-8 22814028

[143] Fitzgerald P Cassidy Eugene M Clarke G Scully P Barry S Quigley Eamonn MM Tryptophan catabolism in females with irritable bowel syndrome: relationship to interferon-gamma, severity of symptoms and psychiatric co-morbidity. Neurogastroenterol Motil (2008) 20(12):1291–7. 10.1111/j.1365-2982.2008.01195.x 18823288

[144] Arvidson NG Larsson A Larsen A. Disease activity in rheumatoid arthritis: fibrinogen is superior to the erythrocyte sedimentation rate. Scand J Clin Lab Invest (2002) 62(4):315–9. 10.1080/003655102760145889 12476931

[145] Parada-Turska J Rzeski W Zgrajka W Majdan M Kandefer-Szerszen M Turski W. Kynurenic acid, an endogenous constituent of rheumatoid arthritis synovial fluid, inhibits proliferation of synoviocytes in vitro. Rheumatol Int (2006) 26(5):422–6. 10.1007/s00296-005-0057-4 16220290

[146] Opitz CA Litzenburger UM Sahm F Ott M Tritschler I Trump S An endogenous tumour-promoting ligand of the human aryl hydrocarbon receptor. Nature (2011) 478(7368):197–203. 10.1038/nature10491 21976023

[147] Dantzer R O’Connor JC Lawson MA Kelley KW. Inflammation-associated depression: from serotonin to kynurenine. Psychoneuroendocrinology (2011) 36(3):426–36. 10.1016/j.psyneuen.2010.09.012 21041030 PMC3053088

[148] Cruz-Haces M Tang J Acosta G Fernandez J Shi R. Pathological correlations between traumatic brain injury and chronic neurodegenerative diseases. Transl Neurodegener (2017) 6:20. 10.1186/s40035-017-0088-2 28702179 PMC5504572

[149] Fukui S Schwarcz R Rapoport SI Takada Y Smith QR Blood-brain barrier transport of kynurenines: implications for brain synthesis and metabolism. J Neurochem (1991) 56(6):2007–17. 10.1111/j.1471-4159.1991.tb03460.x 1827495

[150] Jauch D Urbanska EM Guidetti P Bird ED Vonsattel JP Whetsell WO Jr Dysfunction of brain kynurenic acid metabolism in Huntington’s disease: focus on kynurenine aminotransferases. J Neurol Sci (1995) 130(1):39–47. 10.1016/0022-510X(94)00280-2 7650530

[151] Wilcock DM Griffin WST Down’s syndrome, neuroinflammation, and Alzheimer neuropathogenesis. J Neuroinflammation (2013) 10:84 10.1186/1742-2094-10-84 23866266 PMC3750399

[152] Turski MP Turska M Paluszkiewicz P Parada-Turska J Oxenkrug GF. Kynurenic acid in the digestive system-new facts, new challenges. Int J Tryptophan Res (2013) 6:47–55. 10.4137/IJTR.S12536 24049450 PMC3772988

[153] Kuc D Zgrajka W Parada-Turska J Urbanik-Sypniewska T Turski WA. Micromolar concentration of kynurenic acid in rat small intestine. Amino Acids (2008) 35(2):503–5. 10.1007/s00726-007-0631-z 18235993

[154] Paluszkiewicz P Zgrajka W Saran T Schabowski J Piedra JL Fedkiv O High concentration of kynurenic acid in bile and pancreatic juice. Amino Acids (2009) 37(4):637–41. 10.1007/s00726-008-0183-x 18836681

[155] Turski MP Turska M Zgrajka W Kuc D Turski WA. Presence of kynurenic acid in food and honeybee products. Amino Acids (2009) 36(1):75–80. 10.1007/s00726-008-0031-z 18231708

[156] Turski MP Turska M Kocki T Turski WA Paluszkiewicz P Kynurenic acid content in selected culinary herbs and spices. J Chem (2015) 2015:6 10.1155/2015/617571

[157] Radhika V Dhanasekaran N Transforming G proteins. Oncogene (2001) 20(13):1607–14. 10.1038/sj.onc.1204274 11313908

[158] Cooper AJL The role of glutamine transaminase K (GTK) in sulfur and α-keto acid metabolism in the brain, and in the possible bioactivation of neurotoxicants. Neurochem Int (2004) 44(8):557–77. 10.1016/j.neuint.2003.12.002 15016471

[159] Desbonnet L Garrett L Clarke G Bienenstock J Dinan TG. The probiotic *Bifidobacteria infantis*: an assessment of potential antidepressant properties in the rat. J Psychiatr Res (2008) 43(2):164–74. 10.1016/j.jpsychires.2008.03.009 18456279

[160] Dolecka J Urbanik-Sypniewska T Skrzydło-Radomańska B Parada-Turska J. Effect of kynurenic acid on the viability of probiotics in vitro. Pharmacol Rep (2011) 63(2):548–51. 10.1016/S1734-1140(11)70522-9 21602611

[161] Kaczorek E Szarek J Mikiewicz M Terech-Majewska E Schulz P Malaczewska J Effect of feed supplementation with kynurenic acid on the morphology of the liver, kidney and gills in rainbow trout (*Oncorhynchus mykiss* Walbaum, 1792), healthy and experimentally infected with *Yersinia ruckeri*. J Fish Dis (2017) 40(7):873–84. 10.1111/jfd.12567 27690267

